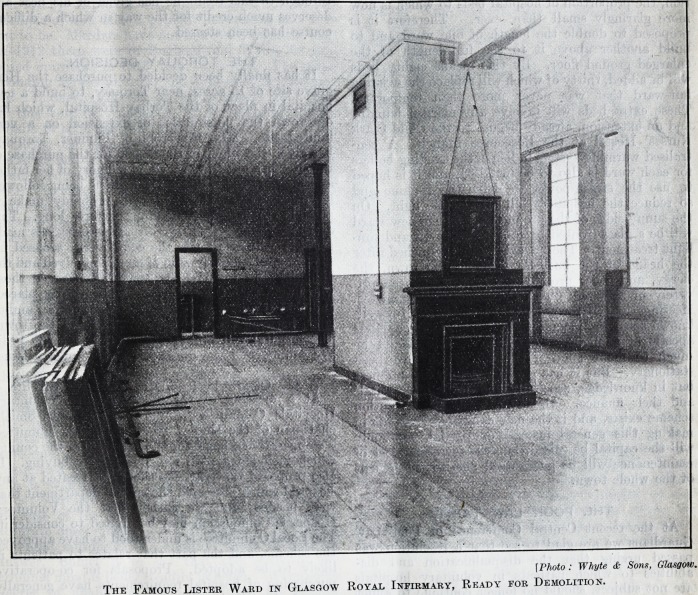# Hospital Progress and Finance

**Published:** 1924-04

**Authors:** 


### HOSPITAL PROGRESS AND FINANCE.

### THE FATE OF THE LISTER WARD.

By the time that these lines are in print, it seems that the Lister Ward at the Glasgow Royal Infirmary will have been destroyed, and the managers, in face of the public opinion of the world, will have added a new act of vandalism to history. By the beginning of March the block, of which the Lister Ward occupied the ground floor, was in the hands of the house-breakers, and, unless an unforeseen repentance causes their hands to be stayed at the eleventh hour, the Lister Ward will have been converted into a rubbish heap. The Scottish Office was appealed to in vain, because it has no power in the matter, and the only lesson to be learnt from this lamentable affair is the necessity for protecting public monuments by a public authority. The action of the Glasgow managers shows that the immediate trustees of such buildings are not always to be trusted, and may, indeed, be the most dangerous enemies that they have to fear. When the approaching Lister centenary arrives we hope that the managers responsible for this policy of destruction, which is all but perpetuated as we write, will have the decency to take no part in the proceedings.

### THE PETERBOROUGH PROPOSAL.

At length it seems likely that Peterborough will have the new hospital which has been contemplated and discussed so often. At all events, a meeting of the general committee of the Peterborough War Memorial New Hospital has accepted the recommendations of the Hospital Joint Committee, whose report, at the time of writing, still awaits the decision of the governors of Peterborough Infirmary. The Joint Committee, after weighing the possibility of extending the old infirmary, and the financial condition, present and future, of the New Hospital scheme, unanimously recommend that the new War Memorial scheme should go forward. They find the old infirmary incapable of adequate extension, and approve the site and possibilities presented by Mr. Bunting. The cost, excluding the site, is expected to be £68,000, and for £45,000 a preliminary part, with more beds than are at present available, could be built. Including the preparation of plans and securing of tenders, three years would be required to complete the proposed scheme. At present £30,000 is in hand, and by the beginning of 1925, £44,000 should be available. This report has also been sent

[*Photo: Whyte & Sons, Glasgow.*

THE FAMOUS LISTER WARD IN GLASGOW ROYAL INFIRMARY, READY FOR DEMOLITION.

to the Infirmary, whose separate decision must be recorded before the final prospects can be judged. In so far as the Infirmary favoured the Joint Committee, it must be considered to have promised sympathetic attention to their recommendations, so that the scheme has now been defined, and has consequently reached an interesting stage.

### THE BOURNEMOUTH CAMPAIGN.

To overtake arrears of building and repairs since 1914, the Bournemouth hospitals, which are now out of debt, are appealing for £50,000. If their needs have grown in the meantime so has the local population, the proportion of hospital beds to which is now more glaringly small than ever. Therefore it is proposed to double the length of one ward and to build another above it to the full length of the enlarged ground floor. By this means eighty beds will be added, thirty of which will replace the existing hut-ward that was never more than temporary. These extra beds will involve an enlarged laundry and an extension, already begun, to the Child Clark Nurses' Hostel at the Boscombe branch. A centralised warming system in lieu of the existing boilers for each ward is also to be installed, and it is hoped to use the steam-power to drive the dynamo and so reduce the hospital's bill for electric light. On the sunny sides of the ground floor of the new ward will be a glass-fronted balcony for open-air and sunlight treatment. The appeal is being launched after the heads of the business firms in the town, the clergy, and other prominent people have been privately in conference with the hospitals' committee. Meetings in the town are to be addressed by well-known speakers, including Dr. Saleeby, that medical apostle of sunlight. Ten years' arrears would be much to make good if the population had been stationary. But it has grown not only in numbers but in knowledge, especially knowledge of hospitals and their finance. Already a weekly contribution scheme exists, and if the new campaign succeeds in making this general instead of partial, not only will the capital be raised, but the increased cost of maintenance will be guaranteed also to the benefit of the whole town.

### THE POOR LAW TAINT.

At the recent Central Conference of Poor Law Guardians we are glad to see that a resolution was passed urging that the disqualification and disabilities to which patients in voluntary hospitals are not subject, should no longer continue to afflict paying patients in Poor Law Infirmaries. It is idle to pretend that no " taint " any longer exists when self-supporting patients suffer legal disabilities. When these are removed the " taint " will disappear, for these constitute the " taint " that keeps people away from these otherwise excellent public hospitals.

### MR. WAKE'S RETIREMENT.

Mr. Philip K. Wake, the distinguished chairman of the Sheffield Royal Hospital, is retiring after many years' service. He joined the Board thirty years ago, and two years later became the hospital's treasurer, a post which he retained till 1907, when he was elected to the chairmanship. During his period of service the hospital has been rebuilt at a cost of £100,000, and a new nurses' home has been established in Eldon Street. Last autumn three new wards were opened, and the hospital has a total of 316 beds, if those at the annexe are included. Chairmanship of a Sheffield hospital has been no light task during the past thirty years, for finance has been a severe difficulty, so severe that the Sheffield Hospitals' Fund had to be created to meet the difficulty. This is now a well-known success, and Mr. Wake will retire feeling that not only his institution, but the voluntary system in Sheffield, is stronger and more adequate than it has been at any time. In winning through to this result he has given of his best and deserves much credit for the way in which a difficult course has been steered.

### THE TORQUAY DECISION.

It has finally been decided to purchase the Hengrave site of 15 acres, near Torquay, to build a new hospital in place of the Torbay Hospital, which has outgrown the possibilities of extension on a very limited site. Mrs. Rowcroft, of Pilmuer, Torquay, has given the whole of the £8,000 for the purchase of the site, as a gift to the town in memory of her father and mother. This magnificent gift is being followed by other donations, which will probably make it possible to consider building before long. This decision, which Mrs. Rowcroft's generosity has made possible will also settle the controversy aroused by the proposal, since the sum of money involved and the difficulty anticipated in raising it encouraged those who urged that the site was inconveniently placed, and that it might also involve the cost of an ambulance between the proposed hospital and the town. The hospital will probably contain about 200 beds. We summarised the question in our January issue.

### A CO-OPERATIVE STEP IN WORCESTER.

At a meeting of the Worcestershire Voluntary Hospitals Committee, Capt. T. S. Stewart-Smith, chairman of the Dudley Guest Hospital, submitted a co-operative scheme for the hospitals of the county which included a plan for co-operative buying. It also proposed that patients should be treated at any hospital which possessed the special department that they needed, and we gather that the Voluntary Hospitals Commission may be invited to consider it. The Local Committee is understood to have approved the scheme, and the part that relates to patients is likely to be adopted. Proposals for co-operative purchasing of hospital requirements have generally proved abortive in the past, but there are already hospitals which find it possible to dispense in some of their purchases with contracts, and to buy wholesale without apparently offending local tradesmen. As this plan involves a man with a gift for making the most of the markets, the success of the plan seems to depend upon some such person being found in every hospital group, since he could do better for several hospitals and with orders of larger bulk than for one institution. When such a man is found, it should also be possible to avoid friction with local tradesmen by allotting to them any class of goods that it is hardly worth while to buy in the wholesale market. We await the details of Capt. Stewart-Smith's proposals with interest and curiosity.

## Figures and Tables

**Figure f1:**